# Mineralocorticoid Receptor Antagonists Therapy in Resistant Hypertension: Time to Implement Guidelines!

**DOI:** 10.3389/fcvm.2015.00003

**Published:** 2015-02-04

**Authors:** Giuseppe Maiolino, Matteo Azzolini, Gian Paolo Rossi

**Affiliations:** ^1^Department of Medicine (DIMED), Internal Medicine 4, University of Padova, Padova, Italy

**Keywords:** mineralocorticoid receptor antagonists, pathogenesis, resistant hypertension, therapy, review

## Abstract

Despite the availability of anti-hypertensive medications with increasing efficacy up to 50% of hypertensive patients have blood pressure levels (BP) not at the goals set by international societies. Some of these patients are either not optimally treated or are non-adherent to the prescribed drugs. However, a proportion, despite adequate treatment, have resistant hypertension (RH), which represents an important problem in that it is associated to an excess risk of cardiovascular events. Notwithstanding a complex pathogenesis, an abundance of data suggests a key contribution for the mineralocorticoid receptor (MR) in RH, thus fostering a potential role for its antagonists in RH. Based on these premises randomized clinical trials aimed at testing the efficacy of MR antagonists (MRAs) in RH patients have been completed. Overall, they demonstrated the efficacy of MRAs in reducing BP and surrogate markers of target organ damage, such as microalbuminuria, either compared to placebo or to other drugs. In summary, owing to the key role of the MR in the pathogenesis of RH and on the proven efficacy of MRAs we advocate their inclusion as an essential component of therapy in patients with presumed RH. Conversely, we propose that RH should be diagnosed only in patients whose BP values show to be resistant to an up-titrated dose of these drugs.

## Resistant Hypertension: Wandering Definitions are not Helpful

The development of novel anti-hypertensive medications with increasing efficacy and decreasing adverse effects might generate the deceitful impression that decreasing patients’ blood pressure (BP) at the goals set by international societies is no longer an issue. By contrast, available data indicate just the opposite, as recently confirmed by an analysis of the National Health and Nutrition Examination Survey (NHANES) showing that only 53% of treated hypertensive participants had BP at goal (1), as well as by a cross-sectional analysis of the Framingham Heart Study and a recent study including 172,432 subjects in which only from 48 to 64% of treated patients had BP at goal (2, 3). While differences in these rates (7.6–18%) likely reflect several factors, including, above all, the stringency of the criteria used (4, 5), it is clear that not all of the hypertensive patients with uncontrolled BP values have resistant hypertension (RH) and represent therapeutic failures. Good deals of them are either not optimally treated (6) or are non-adherent to the prescribed drugs (7–9) or are taking medications that can raise BP [see for review Rossi et al. (10)]. Therefore, they should be more appropriately considered medical failures. However, the subset of patients that have RH according to the definitions of the international societies (Table 1) represent an important medical problem as they are exposed to progressive damage in the target organs (11) and thereby to an excess risk of cardiovascular events as recently reemphasized by a *post hoc* analysis of the ALLHAT database (12).

**Table 1:** **Definitions of resistant hypertension according to major scientific societies**.

Society/year/reference	Definition
Seventh JNC 2003 (16)	Blood pressure above goal
	Adhesion to full doses of an appropriate three-drug regimen
	One of the three agents is necessarily a diuretic
AHA Scientific Statement 2008 (13)	Blood pressure above goal in spite of the concurrent use of three antihypertensive agents of different classes
	One of the three agents ideally should be a diuretic
	Patients whose blood pressure is controlled but requires four or more medications
NICE Guidelines 2011 (15)	Blood pressure higher than 140/90 mmHg after treatment with optimal or best tolerated doses of drugs
	Drug therapy including an ACE inhibitor or an ARB combined with a calcium channel blocker and a diuretic
ESH/ESC Guidelines 2013 (14)	Systolic and diastolic blood pressure values above 140 and 90 mmHg, respectively
	Therapeutic strategy including lifestyle measures
	Drug therapy including a diuretic and two other antihypertensive drugs belonging to different classes at adequate doses

All the current guidelines define RH, albeit with slight differences (Table 1), by the inability to lower BP levels at goal with a treatment including at least three drugs of different classes, one being ideally (13) or necessarily (14–16) a diuretic, all prescribed at optimal doses. The American Heart Association (AHA) definition is even less restrictive in that it defines as RH patients those on more than three medications, even though their BP is at target (13). It is worth to underline that only the European Society of Hypertension (ESH) guidelines pay heed to the implementation of appropriate lifestyle changes (14), a treatment option neglected in the others (13, 15, 16). The measures associated to a better BP control are: (1) salt restriction [systolic and diastolic BP reduction of 6/3 mmHg up to 20/10 mmHg, respectively (17)]; (2) diet reduced on saturated fat/cholesterol and increased on vegetables, fruits, and fish [5/3 mmHg of systolic and diastolic BP reduction, respectively (18)]; (3) weight reduction [1 mmHg of BP decrease per kilogram of weight loss (19)]; (4) aerobic physical exercise [7/5 mmHg of systolic and diastolic BP reduction, respectively (20)]; (5) limitation of alcohol consumption [1.9/0.6 mmHg of systolic and diastolic BP reduction, respectively (21)]; (6) smoking cessation (22) (Table 2). The highlighting of lifestyle measures by the ESH guidelines is commendable, but should be interpreted as appropriate counseling given to patients. Nonetheless, strict application of this definition implies that subjects non-compliant with these directions, just like those non-compliant to medical treatment, would not meet the criteria for RH.

**Table 2:** **Lifestyle changes associated to a BP decrease**.

Salt restriction (6/3 mmHg up to 20/10 mmHg of systolic and diastolic BP reduction, respectively)
Diet low on saturated fat/cholesterol and high on vegetables, fruits, fish diet (5/3 mmHg of systolic and diastolic BP reduction, respectively)
Weight reduction (1 mmHg of BP decrease per kilogram of weight loss)
Aerobic physical exercise (7/5 mmHg of systolic and diastolic BP reduction, respectively)
Limitation of alcohol consumption (1.9/0.6 mmHg of systolic and diastolic BP reduction, respectively)
Smoking cessation

The three major international societies (13, 14, 16) do not specify the drugs in their definition of RH, while the British NICE dictates the classes of medications, which are an angiotensin converting enzyme inhibitor (ACE-I) or an angiotensin II receptor blocker (ARB), a calcium channel blocker (CCB), and a diuretic (15). Messerli et al. proposed an even more restrictive definition: a systolic BP of 160 mmHg or higher despite treatment with a full dose of a renin–angiotensin–aldosterone system (RAAS) blocker (either an ACE-I, an ARB, or a renin inhibitor), a CCB (either dihydropyridine or non-dihydropyridine), a diuretic (preferentially chlorthalidone), and, if tolerated, a mineralocorticoid receptor (MR) antagonist (MRA) (spironolactone or eplerenone) (23).

Such more strict criteria to define RH could be useful but carry some limitations: patients not having BP at goal despite being treated with more than three drugs, but with intolerance or contraindication to a class of drugs, such as, for example, a RAAS blocker in subjects with bilateral renal artery stenosis, or a CCB in those with low ejection fraction heart failure, would not meet the definition for RH. Such incorrect classification of these patients, can imply that they will not be perceived as being at high-risk, thus affecting their management and prognosis and impairing data comparability of cohort studies or clinical trials using different definitions.

In summary, there is an urgent need to homogenize the definitions of RH by including also the patients intolerant to the drugs suggested by guidelines and experts (15, 23) if in need of more than three drugs and with BP above goal.

## Resistant Hypertension: Relevance of the Issue

Data from large clinical trials of anti-hypertensive therapy suggest that the prevalence of RH can be as high as 35% (24–29) (Table 3). These numbers are probably overestimated since patients enrolled in these trials entail selected cohorts with risk profile and comorbidities higher than the ordinary hypertensive population.

**Table 3:** **Resistant hypertension prevalence in randomized controlled trials**.

Study	Pts (n°)	Hypertensive Pts characteristics	Definition RH	Prevalence of RH (%)
ALLHAT	14,684	≥55 y/o	Pts on ≥3 drug classes BP ≥140/90 mmHg OR pts ≥4 drug classes	12.7
		stage 1 or 2 HTN	
		≥1 RF for CAD	
ASCOT-BPLA	19,257	40–79 y/o	Pts on ≥3 drug classes BP ≥140/90 mmHg OR pts ≥4 drug classes	48.5
		≥3 CV RF	
INVEST	22,576	≥55 y/o	Pts on ≥3 drug classes (HCT included) BP ≥140/90 mmHg	28.8
		documented CAD	
LIFE	9,222	55–80 y/o	Pts on ≥3 drug classes (HCT included) BP ≥140/90 mmHg	53.9
		EKG signs LVH	
CONVINCE	16,476	≥55 y/o	Pts on ≥3 drug classes (HCT included) BP ≥140/90 mmHg	34.3
		≥1 CV RF	

*BP, blood pressure; CAD, coronary artery disease; CV, cardiovascular; EKG, electrocardiogram; HCT, hydrochlorothiazide; HTN, hypertension; LVH, left ventricular hypertrophy; Pts, patients; RF, risk factor; RH, resistant hypertension; y/o, years old*.

Observational studies likely provide a more genuine estimate of the actual figures involved and show that RH involves 10–20% of the general population of hypertensive patients (30). However, some drawbacks mandate caution in interpreting these data. For instance, in a United States cohort, after exclusion of non-adherent subjects, who can entail 37% of patients with uncontrolled hypertension (8), a rate of RH of 12.8% was reported. However, the assumption of diuretics, which for most guidelines represents a *condicio sine qua non-for* the diagnosis, was neglected (31). Moreover, patients with white-coat syndrome, who can be up to 40% of patients with “resistant hypertension” (4), were not excluded. In another US study, Daugherty et al. found that the prevalence of RH was 16.2%, but the same biases existed (32). Finally, a Spanish study that estimated a prevalence of RH of 8.9% and devoted proper attention to exclude those with the white-coat effect did not assess drug adherence (4). Of interest, two studies looking specifically at the rate of RH provided quite different estimates. According to Pierdomenico et al., who defined RH as office BP ≥140 or ≥90 mmHg for systolic and diastolic, respectively, at least at two visits while on triple therapy, the prevalence would be 18% (5). By contrast, the Spanish ambulatory blood pressure monitoring (ABPM) Registry that in similarly treated patients based the definition on identical criteria for clinical BP but also used ABPM daytime BP ≥130 or ≥80 mmHg for systolic and diastolic, respectively, reported a prevalence of 7.6% (4). Hence, it is altogether evident that ABPM is necessary to pinpoint those with clinic high BP that is due to the white-coat phenomenon.

The attention that RH is receiving mainly derives from the evidence that it associates not only with subclinical target organ damage, such as left ventricular hypertrophy (11, 33, 34), microalbuminuria (31, 33–36), impaired renal function (31, 34), and vascular involvement revealed by carotid intima media thickening (11) exceeding that of patients with well controlled BP, but also with a worse prognosis. These subjects are in fact exposed to an excess risk of stroke, myocardial infarction, congestive heart failure, and chronic kidney disease (12, 37). Indeed, while studies comparing resistant and non-resistant hypertensives consistently showed a higher risk in former, up to 50% (hazard ratio 1.47, 95% confidence interval 1.33–1.62) of cardiovascular events and renal events (5, 32, 38), the estimates of this excess risk are imprecisely known. For example, in a survey of more than 50,000 hypertensive patients with at least three cardiovascular risk factors the detrimental effect was lower than expected, with an excess risk for cardiovascular events (hazard ratio 1.18, 95% confidence interval 1.10–1.26), especially non-fatal stroke (hazard ratio 1.26, 95% confidence interval 1.10–1.45) and congestive heart failure (hazard ratio 1.36, 95% confidence interval 1.23–1.51) in patients with RH compared to non-resistant hypertensives (39).

Thus, even though the evidence collectively indicates that RH implies an excess risk of cardiovascular events, the extent of this increased risk varies widely, likely reflecting the variable definitions of RH across studies.

## Pathogenesis of Resistant Hypertension and Potential Benefits of Mineralocorticoid Receptor Antagonists

In patients with uncontrolled BP pseudo-resistance must be excluded beforehand. The latter can be secondary to: (1) poor office BP measurement technique, (2) “white-coat” effect, which encompasses up to 40% of patients with uncontrolled BP (4), (3) non-adherence to the prescribed therapy [30–40% of subjects (7, 8)], or (4) a suboptimal anti-hypertensive regimen, owed to inappropriate drug associations or therapeutic inertia (40–42). Only after exclusion of pseudo-resistance and of secondary hypertension patients can be labeled as having RH, whose most common causes are: excessive salt intake and obesity. In our view, the diagnosis of RH should be regarded as a provisional classification of the patient and by no means a long-time definition for the following reason: many patients with RH if properly investigated are found to be affected by secondary forms of high BP.

Several substances or pharmacological agents can induce hypertension or reduce the efficacy of anti-hypertensive therapies and have been associated to RH (10). A special mention among the pharmacological agents pertains to the non-steroidal anti-inflammatory, oestro-progestinic, steroid, and immunosuppressive drugs, because of their widespread use. Likewise, due to their increasing diffusion a careful history on abuse substances, such as cocaine and amphetamines, as well as alcohol and coffee, should be elicited.

Compared to uncomplicated well controlled hypertensive patients those with uncontrolled BP display a higher prevalence of secondary hypertension, due to primary aldosteronism (7–20%) (43, 44), renal artery stenosis (2–24%) (45, 46), and chronic kidney diseases (30–40%) (31, 34, 36, 47), with rates varying across studies because of the different selection criteria, cohorts, and diagnostic work-up exploited.

Primary aldosteronism is the most frequent cause of secondary hypertension (48, 49) in newly diagnosed referred consecutive hypertensive patients and when surgically non-curable forms are present its most appropriate treatment are MRAs. In the PAPY study, including 1,125 consecutive hypertensive patients enrolled at 18 referral centers throughout Italy, who were screened while either on pharmacological wash-out or on CCB and/or doxazosin treatment, the prevalence of primary hyperaldosteronism was 11.2 and 43% of these cases had an aldosterone-producing adenoma (APA) (49). Of note, only 48% of patients with APA and 17% of those with idiopathic hyperaldosteronism had hypokalemia at clinical presentation. Moreover, even though the prevalence of primary aldosteronism increased with the severity of hypertension most cases were seen in stage I and stage II hypertension. These evidences contradict the fallacious perception that a diagnosis of primary aldosteronism should be pursued only in patients with hypokalemia (49). Because of the misconception that primary aldosteronism is a disease of hypertensive patients with hypokalemia and severe/resistant hypertension it goes mostly unnoticed (49). Furthermore, the diagnostic work-up bears more difficulties in patients on multiple drugs, most of which affect the RAAS (50).

In addition to primary aldosteronism, most patients with RH develop a secondary aldosteronism triggered by a diuretic-induced sodium depletion, which activates the RAAS, similarly to what happens with sodium restriction (17, 51). In this context, it is worth noting how MRAs can effectively counteract this effect and improve BP control. Similarly, long-term use of RAAS inhibitors (ACE-I and ARB) is known to be associated with angiotensin II increase and ensuing aldosterone production, which can contribute to resistance to the anti-hypertensive therapy. In fact, whereas the acute effect of RAAS inhibitors is a decrease of aldosterone, in the long run aldosterone can increase to levels even higher than pretreatment (52, 53), as well documented in a relevant proportion (10–53%) of the patients with heart failure or chronic kidney disease on protracted ACE-I or ARB treatment (54).

The MR, besides its well-known effects in the kidney, has extra-renal actions that could affect BP regulation (55), including activation of the sympathetic nervous system (56), endothelial dysfunction (57), and vasoconstriction (58) through stimulation of the human vascular smooth muscle cells (59). Moreover, blacks have lower plasma renin activity (PRA) and plasma aldosterone levels than whites, but their BP is directly associated with the plasma aldosterone concentration and increases with 9-α fludrocortisone administration, an effect that does not occur in whites (60). Hence, collectively these observations suggest differences in the individual sensitivity of the vasculature to MR activation (61).

Finally, the activity of the MR-dependent pathways can be triggered without increases of plasma aldosterone levels through mechanisms different from raised receptor sensitivity, such as increased receptor expression or stimulation by other ligands [reviewed in Ref. (62)], as for example angiotensin II (63–65) or cortisol (62).

Excessive dietary salt intake with ensuing volume overload is a well recognized risk factor for RH (66, 67) owing to its pressor effect and its blunting of the BP-lowering action of anti-hypertensive agents. According to the available studies 90% of the patients with RH have some degree of plasma volume expansion (68) and raised levels of brain-type and atrial natriuretic peptides (69). The same studies also showed high levels of plasma aldosterone and aldosterone–renin ratio, and low PRA, which could be explained either by the high prevalence of undetected primary aldosteronism (43, 44) or by the secondary aldosteronism due to diuretic treatment (69). Two different approaches relying on this premises proved to be efficacious in RH patients: (1) the estimation of body volume expansion by measurement of thoracic bioimpedance as a guide to up-titrate diuretics (70); (2) the sequential blockade of the nephron by means of stepped addition of four low-dose diuretics, spironolactone among others, to antagonize the sodium and water reabsorption along the nephron (51).

Obesity (50–55%) is common in patients with RH (4, 34) and is associated to suboptimal BP control as demonstrated by the HYDRA study (71). The underlying pathophysiology of the BP elevation in obese patients entails a combination of sodium retention, activation of the sympathetic nervous system (72), sleep-related breathing disorders, and relative hyperaldosteronism with ensuing volume expansion (73).

Patients with RH bear an extensive, up to 70%, prevalence of obstructive sleep apnea (OSA) (45, 74–76), which is increasingly recognized as an important determinant of uncontrolled hypertension. The improvements of BP control in affected subjects undergoing efficacious treatment of OSA with continuous positive air pressure ventilation support a causal link between these conditions (77, 78). The putative pathogenesis is possibly ascribed to the increased upper airway resistance and intermittent hypoxia, which activate the sympathetic nervous system and the RAAS, as suggested by the association of its severity with plasma aldosterone levels (76) and by the improvement of OSA patients on spironolactone treatment (79).

Taken together these evidences reveal the complexity of the pathogenesis of RH and a key role for the MR in it, thus suggesting the relevant role of MRA therapy in this field.

## Current Therapies for Resistant Hypertension

Adherence to a low-sodium diet ranks first among the lifestyle measures to be recommended to all hypertensive patients because it is highly effective in decreasing both systolic and diastolic BP (4–7 and 1–3 mmHg, respectively) as recently demonstrated (47, 80, 81) with effects even more striking in patients with RH (67). Moreover, regular isotonic exercise should be included in the therapeutic approach to RH in that it is able to decrease BP even in subjects with low responsiveness to medical treatment (82).

Another point that has to be highlighted and pursued in RH is that drug association therapy is typically more effective than increasing the dose of each medication. This was clearly evidenced in a meta-analysis of 354 randomized placebo-controlled trials showing that doubling the anti-hypertensive agent dosage was less effective in lowering systolic and diastolic BP than combination therapy [2 and 1 mmHg vs. 6–7 and 3–4 mmHg, respectively (83)]. These results were later confirmed by a subsequent meta-analysis (84) and in the OSCAR trial examining an intensified monotherapy regimen with a high-dose ARB (40 mg olmesartan) and an association approach with a low-dose ARB (20 mg olmesartan) plus a dihydropiridinic CCB (amlodipine or azelnidipine) (85). Despite a similar decrease of BP in the two treatment arms, combination therapy reduced the incidence of cardiovascular events and death in high-risk patients with a history of cardiovascular morbidity at baseline.

Among diuretics, chlorthalidone, a long-acting thiazide-like diuretic, is held to be more potent than hydrochlorothiazide in lowering BP (86) and therefore should be preferred, according to Messerli, over the latter, which at its usual dose of 12.5–25 mg is inferior to other anti-hypertensive agents (87, 88). However, due to its long half-life (50 h), which exposes to a carry-over effect with daily assumption, it confers a higher risk of hypokalemia. The chlorthalidone-induced hypokalemia was in fact suggested to be a sign of undetected primary aldosteronism (89).

The ESH/ESC guidelines suggest that patients with RH and persistently elevated BP values despite medical treatment optimization should be considered for invasive procedures such as carotid baroreceptor stimulation and renal denervation (see Table 4). The former seems a promising technique, as evidenced by the Rheos pivotal trial. This relatively large randomized controlled trial showed a borderline significant greater SBP reduction in the treatment arm compared to the placebo group (16 vs. 9 mmHg, respectively, *p* = 0.08) (90). Regarding renal denervation, the high expectations generated by the SYMPLICITY HTN-1 (91) and - 2 (92) trials were attenuated by the results of the SIMPLICITY HTN-3 trial, which could not demonstrate an advantage of the procedure compared to sham controls in patients with RH in part due to the larger than expected BP fall in the sham group, but, interestingly enough, also for the higher rate of treatment with MRAs in this trial than in the previous ones (93).

**Table 4:** **ESH/ESC guidelines on resistant hypertension invasive treatment**.

Recommendation	Class	Level
Invasive procedures may be considered in RH patients if drug treatment is ineffective	IIb	C
Invasive procedures should be carried out by experienced operators; diagnosis and follow-up should be restricted to hypertension centers	I	C
Invasive procedures should be considered only in truly RH patients with clinic SBD ≥160 mmHg and DBP ≥110 mmHg and confirmed at ABPM	I	C

*ABPM, ambulatory blood pressure monitoring; DBP, diastolic blood pressure; RH, resistant hypertension; SBP, systolic blood pressure*.

In conclusion, although in the definition of RH only the generic term “diuretic” is mentioned, by no means all diuretics are equal and a careful choice of the agent along with appropriate up-titration of the dose(s) are key for bringing BP under control. Among diuretics, MRAs deserve a special place for the multitude of potential benefits they provide, so that some experts advocate their use as a fourth line add-on drug in patients with RH (23, 94).

## Evidences that Mineralocorticoid Receptor Antagonists are Efficacious in Resistant Hypertension

Treatment of hypertension with MRAs was introduced almost 40 years ago (95–97). Spironolactone proved to be as effective as propranolol (95) and chlorthalidone (97), and remained efficacious as an add-on therapy in patients already receiving a diuretic (98), probably due to the blunting of the aldosterone breakthrough effect. Eplerenone was developed in an attempt to overcome the adverse effects of MRAs, including erectile dysfunction and gynecomastia, which depends on their anti-androgenic effects (99–101). This compound was suggested to be more selective (102) and at least as effective as losartan (103) or even superior to the former in patients with low-renin hypertension (104) and in those of African-American descent (103). Eplerenone was also shown to be as effective as enalapril (105, 106) and amlodipine (107) as an add-on therapy to ACE-I or ARB monotherapy (108). However, it is shorter acting and less potent than spironolactone, canrenone, and potassium canreonate. Moreover, when used at doses equipotent as spironolactone on BP, it was found to cause estrogen-like effects. Of note, the EVALUATE study, a multicenter, randomized, double blind clinical trial of patients with hypertension and stage 2 and 3 chronic kidney disease, showed that both BP and microalbuminuria were significantly lower over 52 weeks of follow-up in patients assigned to a low-dose eplerenone once a day than in those receiving placebo, both on top of treatment with an ACE-I or an ARB. No hyperkalemia, gynecomastia, or erectile dysfunction were observed with such a low-dose of this MRA (109).

Several authors have highlighted the value of MRAs in patients with RH by virtue of cohort studies (110–116) [reviewed by Ref. (117, 118)] and of placebo-controlled clinical trials (51, 119–122), which are held to provide harder evidences, as outlined in the following paragraphs (Table 5).

**Table 5:** **Randomized controlled trials comparing mineralocorticoid receptor antagonists vs. placebo in resistant hypertension patients**.

Study	Pts (n°)	Pts characteristics	End points	MRA	Dose mg/day	Control	Follow up weeks	Results
Vaclavík et al.	117	RH pts	Decrease of daytime SBP and DBP on ABPM	Spironolactone	25	Placebo	8	5.4 mmHg decrease of daytime SBP
Abolghasmi et al.	41	RH pts with CKD	n/a	Spironolactone	25–50	Placebo	12	30/8 mmHg SBP and DBP decrease (office BP)
Bobrie et al.	167	RH pts	Non-inferiority of SNB relative to SRASB in reducing daytime ambulatory SBP	Spironolactone	25	Ramipril	12	Significant decrease of home SBP and DBP at 4 w with spironolactone
Oxlund et al.	119	RH pts with type 2 DM	Reduction of daytime SBP and DBP at ABPM	Spironolactone	25	Placebo	16	8.9/3.7 mmHg daytime SBP and DBP decrease (ABPM)
Karns et al.	155	RH pts	Mean sitting SBP of LCI vs. placebo	Eplerenone	100	LCI699, placebo	8	No difference LCI699 vs. placebo; decrease of 14.7/9.4 mmHg, SBP and DBP, with eplerenone (ABPM)

*ABPM, ambulatory blood pressure monitoring; CKD, chronic kidney disease; DBP, diastolic blood pressure; DM, diabetes mellitus; n/a, not available; Pts, patients; RH, resistant hypertension; SBP, systolic blood pressure; SNB, sequential nephron blockade; SRASB, sequential renin–angiotensin system blockade; w, weeks*.

The first placebo-controlled trial testing the efficacy of MRAs in RH was the ASPIRANT, which included 117 patients randomized to 25 mg of spironolactone or placebo and assessed with 24 h ABPM (119). After 8 weeks of treatment the MRA decreased mean daytime systolic BP of 5 mmHg (95% CI 10–0.8 mmHg), e.g., the primary endpoint, and microalbuminuria of 4.4 mg/day, while it did not reduce mean daytime diastolic BP (1 mmHg, 95% CI −4–2 mmHg). This trial was stopped prematurely after reaching the primary endpoint in an *ad interim* analysis. However, 24% of the patients enrolled were found to have primary aldosteronism at further evaluation, which most likely contributed to the favorable results of the study. Moreover, patients with glomerular filtration rate lower than 40 ml/min were excluded from the ASPIRANT trial owing to the potential risks of hyperkalemia.

Therefore, a randomized placebo-controlled trial was performed by Abolghasmi et al. to prove the efficacy of MRA therapy in chronic kidney disease patients (120). The authors randomized 41 patients with chronic kidney disease (glomerular filtration rate between 50 and 25 ml/min) to 25–50 mg/day of spironolactone or placebo and found that at 6 weeks the MRA decreased systolic and diastolic BP of 33 and 13 mmHg, respectively, whereas placebo did not affect BP. It is worth highlighting that patients with secondary hypertension other than chronic kidney disease were excluded from the study and that only one out of 19 cases receiving MRA treatment developed hyperkalemia (>5.5 mmol/l). Despite the relevance of these results some drawbacks ought to be mentioned, such as the use of a subjective way to assess therapeutic efficacy of MRA like office BP in lieu of the more objective ABPM and the lack of details on randomization and blinding procedures.

To test the non-inferiority of a sequential nephron blockade strategy (by means of the sequential addition of spironolactone, furosemide, and amiloride) vs. a sequential renin–angiotensin system blockade (by virtue of add-on ramipril and bisoprolol at increasing doses) Bobrie et al. randomized 167 patients with RH, treated with irbesartan 300 mg/day, hydrochlorothiazide 12.5 mg/day, and amlodipine 5 mg/day (51). The trial demonstrated that the sequential nephron blockade was more efficacious than the renin–angiotensin system blockade at decreasing BP in these patients as assessed by ABPM. Moreover, it showed a significant decrease in systolic and diastolic BP in patients treated with spironolactone 25 mg/day as compared to ramipril 5 mg/day. These results are potentially important for RH patients but carry two main limitations: (i) the trial was not specifically designed to test the efficacy of spironolactone; (ii) when either spironolactone or ramipril were added, patients were not receiving a maximal dose of diuretic and CCB and therefore, strictly speaking, they did not meet the RH definition.

To test the efficacy in lowering mean sitting systolic BP of a new compound, the aldosterone synthase inhibitor LCI699, Karns et al. randomized 155 patients to receive a placebo or the active drug (122). The study failed his primary endpoint in that it could not demonstrate a significant decrease of BP with the aldosterone synthase inhibitor at various doses compared to placebo. However, interestingly enough, it showed that after 8 weeks eplerenone induced a significant decrease of systolic and diastolic BP compared to placebo (14–15 and 9–11 mmHg, respectively) as assessed at ABPM. Despite not being originally designed to compare MRA therapy with placebo, this study is important in that it extends to the whole MRA class the efficacy in RH.

Finally, Oxlund et al. tested in a randomized placebo-controlled trial the effect of a MRA in reducing BP at ABPM in 119 RH patients with type two diabetes mellitus (121). Using ABPM, the study showed that spironolactone at a mean dose of 35 mg/day significantly reduced systolic and diastolic BP by 9 and 4 mmHg, respectively. The MRA treatment was three times more efficacious than placebo both in lowering BP values at target and in decreasing microalbuminuria.

**Figure 1:**
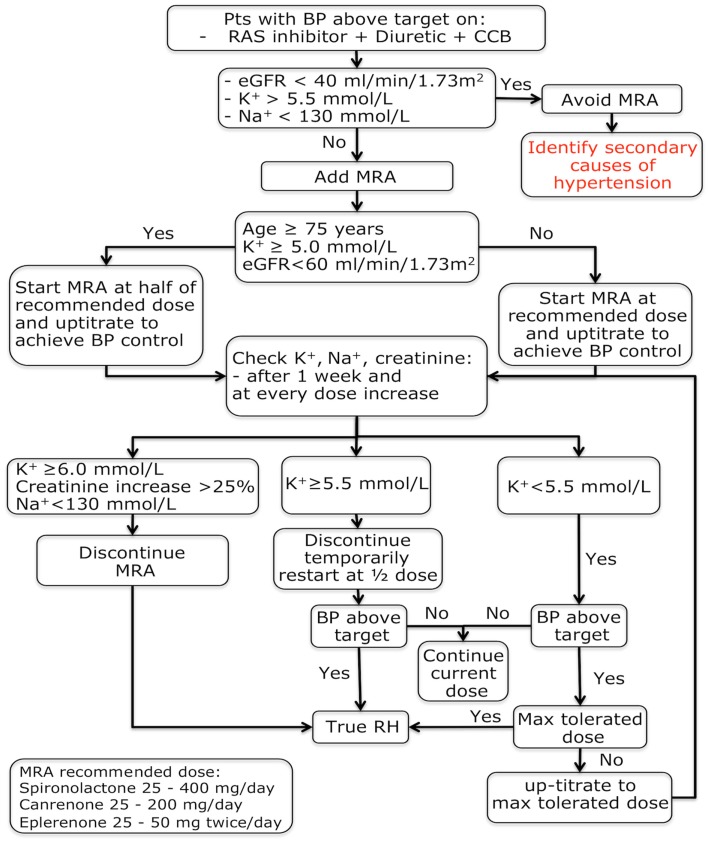
**Mineralocorticoid receptor antagonists treatment algorithm in patients with a provisional resistant hypertension diagnosis**. BP, blood pressure; eGFR, estimated glomerular filtration rate; K^+^, potassium; MRA, mineralocorticoid receptor antagonist; Na^+^, sodium; RH, resistant hypertension.

In summary, both observational studies and randomized trials support the conclusion that MRAs are effective in patients with RH. It has to be acknowledged, however, that these trials had few drawbacks including small sample size (51, 119–122), absence of a systematic exclusion of patients with secondary hypertension (119–122), white-coat hypertension (120, 122), and treatment non-adherence (51, 119–122), and endpoints were often not specifically focused to demonstrate the effect MRAs (51, 122). Despite these limitations, the evidence for efficacy of the MRA in patients with RH now appears to be compelling thus strengthening the proposal both of including MRAs as a cornerstone therapy in patients with difficult-to-control hypertension and the need of lack of response to a MRA as a *condicio sine qua non* for the definition of RH (Figure 1).

## Predictors of Mineralocorticoid Receptor Antagonists Efficacy

The main issue in dealing with RH is how to select the appropriate treatment for most patients, which implies finding some predictors of their efficacy.

In treatment-naïve hypertensive patients (123) and in those on multiple drugs (112, 124) serum potassium values below 4.0–4.5 mmol/l were reported to forecast MRAs efficacy. However, even though low serum potassium could indicate patients with underlying primary or secondary hyperaldosteronism, not all the literature concur with this finding (119).

To predict the response to MRA almost 40 years ago Karlberg et al. observed that spironolactone was more effective in previously untreated patients with low-renin (PRA <1.0 ng/ml/h) essential hypertension (95), a finding subsequently confirmed (123) and extended to patients with RH tested while on anti-hypertensive treatment (119). A correlation was also reported between the aldosterone–renin ratio and MRA efficacy in patients undergoing MRA monotherapy (123) and with RH (119). However, other studies including untreated stage 1 or 2 hypertensives (105), or enrolling subjects with low-renin hypertension (PRA <1.0 ng/ml/h) (104), patients undergoing treatment with eplerenone as an add-on drug to ACE-I or ARB (125), and patients with multi-drug therapy (126) or with RH (111, 123) dispute these findings.

In conclusion, it remains still controversial if renin, aldosterone, and the aldosterone–renin ratio can predict the BP response to MRA in patients already on anti-hypertensive treatment (104, 105, 111, 119, 123, 125, 126). Most likely this is because these measurements are deeply biased by the concomitant anti-hypertensive treatment (125). In keeping with this interpretation, data were more consistent in treatment-naïve patients where these measurements seem to have a role (95, 123). The use of serum potassium level as a predictor of efficacy could be more reliable; however, its usefulness can be affected by the cut-off value of baseline serum potassium chosen in the different studies as a threshold for up-titrating the MRA treatment (94). In fact, adequate dosing of the MRA is a crucial step for increasing the rate of patients’ response to this treatment (55).

## Contraindications to Mineralocorticoid Receptor Antagonists Therapy

Despite being usually well-tolerated MRAs should be cautiously prescribed to special populations. Pregnant and breast-feeding women should avoid MRAs, which cross the placenta, especially in the first trimester, and enter the breast milk, due to their anti-androgenic effect. Monitoring of side effects should be carried out in male subjects, who can complain of erectile dysfunction and gynecomastia.

Due to a higher probability of side effects elderly (≥75 years) and chronic renal disease (glomerular filtration rate <60 ml/min/1.73 m^2^) patients should be prescribed MRAs judiciously. We suggest to halve the starting dose of the drug and to check renal function, serum potassium, and sodium levels after 1 week and after every dose increase. Using these precautions the prescription of MRAs appears safe as demonstrated by trials conducted in patients with chronic renal insufficiency [glomerular filtration rate ≥25–50 ml/min/1.73 m^2^ (109, 120)] where hyperkalemia occurred only sporadically.

Serum potassium testing is mandatory before MRA therapy prescription, in that hyperkalemia is a serious side effect of these drugs, which therefore must not be given to hyperkalemic patients. However, in normokalemic patients with regular testing these medications are safe as showed by clinical trials completed in subjects with RH (119) or chronic kidney disease (109). As a rule of thumb, it is a safe practice to avoid MRAs administration to patients with hyperkalemia (≥5.5 mmol/l) and to decrease its dose when at reassessment the serum potassium increases ≥5.5 mmol/l, while discontinuing indefinitely this therapy if it increases ≥6.0 mmol/l.

Finally, a particular mention deserves the concurrent prescription of MRAs with non-steroidal anti-inflammatory agents because of their extensive use. These agents reduce the anti-hypertensive treatment efficacy and, furthermore, induce a hyporeninemic hypoaldosteronism thus raising the risk of severe and life-threatening hyperkalemia (127).

## Conclusion

Resistant hypertension is an increasingly recognized problem in hypertension treatment owing to its association with a worse prognosis. Based on increasing evidences demonstrating the contribution of aldosterone to its pathogenesis, few uncontrolled studies and randomized clinical trials were completed on this field and demonstrated the beneficial role of the mineralocorticoid receptor antagonists. Therefore, based on these evidences a strong recommendation should be made to advocate their use as a “must” in patients with resistant hypertension.

## Conflict of Interest Statement

The authors declare that the research was conducted in the absence of any commercial or financial relationships that could be construed as a potential conflict of interest.
